# Preliminary study of improving immune tolerance in vivo of bioprosthetic heart valves through a novel antigenic removal method

**DOI:** 10.3389/fbioe.2023.1141247

**Published:** 2023-03-27

**Authors:** Mingzhe Song, Liang Yi, Zhenjie Tang, Xinlong Xie, Yuhong Liu, XiaoKe Qi, Zhenlin Jiang, ZeGuo Chen, Chunyang Chen, QiYing Wu, ZhongShi Wu

**Affiliations:** ^1^ Department of Cardiovascular Surgery, The Second Xiangya Hospital, Central South University, Changsha, Hunan, China; ^2^ Engineering Laboratory of Hunan Province for Cardiovascular Biomaterials, Changsha, China

**Keywords:** biomaterial, ionic detergent, immunogenicity, bioprosthetic heart valves;, decellualrization

## Abstract

The durability of bioprosthetic heart valves is always compromised by the inherent antigenicity of biomaterials. Decellularization has been a promising approach to reducing the immunogenicity of biological valves. However, current methods are insufficient in eliminating all immunogenicity from the biomaterials, necessitating the exploration of novel techniques. In this study, we investigated using a novel detergent, fatty alcohol polyoxyethylene ether sodium sulfate (AES), to remove antigens from bovine pericardium. Our results demonstrated that AES treatment achieved a higher pericardial antigen removal rate than traditional detergent treatments while preserving the mechanical properties and biocompatibility of the biomaterials. Moreover, we observed excellent immune tolerance in the *in vivo* rat model. Overall, our findings suggest that AES treatment is a promising method for preparing biological valves with ideal clinical application prospects.

## 1 Background

Heart valve disease is a severe condition associated with high morbidity and mortality, and valve replacement is a standard treatment for end-stage disease (Yacoub and Takkenberg, 2005). Biological heart valves are preferred over mechanical valves due to several advantages, including the reduced need for anticoagulation and superior hemodynamics (Jana and Lerman, 2015; Chaux et al., 2016). Bioprosthetic heart valves are mainly made of porcine and bovine pericardium (Schoen and Levy, 2005). However, the immunogenicity of heterogeneous tissue materials can result in rejection, which leads to valve degeneration (Cascalho and Platt, 2001; Gates et al., 2017). To mitigate tissue immunogenicity, xenogeneic tissue valves are typically fixed with glutaraldehyde prior to implantation. However, the use of glutaraldehyde presents well-known drawbacks, including residual glutaraldehyde exhibiting cytotoxic effects that hinder host cell attachment, migration, and proliferation, thereby increasing the risk of calcification and tissue fatigue (Human and Zilla, 2001; Manji et al., 2006). Furthermore, glutaraldehyde cross-linked bioprosthetic valve materials are unable to regenerate *in vivo*, which limits their clinical application, particularly for younger patients. (Chang et al., 2002; Manji et al., 2014). Recent research has demonstrated that antigenic blocking following glutaraldehyde fixation is incomplete, leading to chronic graft-specific immune responses and ultimately reducing the lifespan of the valve (Badylak and Gilbert, 2008; Crapo et al., 2011; Thampi et al., 2013; Gates et al., 2017; Kostyunin et al., 2020).

Reducing tissue antigenicity through decellularization is a promising method to improve immune tolerance and achieve better histocompatibility, remodeling, and long-term persistence of heart valve replacements (Zhou et al., 2010; Crapo et al., 2011; Hulsmann et al., 2012; Collatusso et al., 2019). Currently, a variety of reagents, including enzymes (such as trypsin and nuclease) and detergents (such as TritonX-100, sodium dodecyl sulfate (SDS), and others), are utilized for decellularization (Goldstein, 2005; Mirsadraee et al., 2006). Yang et al. (2009) the most common decellularization method involves a combination of detergent and enzyme treatment. However, decellularization with TritonX-100 or other commonly used detergents can significantly damage the extracellular matrix, and residual fragments can trigger an immune response post-implantation (Schenke-Layland et al., 2003; Roosens et al., 2016). Some studies have also shown that histological decellularization alone is insufficient for removing heterogeneous antigens, such as the known transplantation-related antigen α-Gal (Simon et al., 2003; Daly et al., 2009). Given that existing decellularization methods are unable to fully eliminate immunogenicity, improving decellularization methods to reduce antigenicity is critical.

AES is a new type of ionic detergent with minimal toxicity, biocompatibility, and biodegradability, which is widely used in cleaning products and cosmetics (Zoller, 2008; Deng et al., 2016). AES can effectively disintegrate the cell membrane, bind with the hydrophobic part of the membrane protein, and separate it from the membrane, potentially altering the properties of the protein. We hypothesize that AES could be used to reduce the immunogenicity of bovine pericardium. TritonX-100 is a commonly used detergent for decellularization. Although it has been withdrawn from the market in some countries due to its endocrine-disrupting properties (Luo et al., 2020), many studies have shown that it can effectively remove cells when combined with ribozyme (Liao et al., 2008; Roosens et al., 2016).

In this study, we aimed to evaluate the effectiveness of using AES in reducing the immunogenicity of bovine pericardium when compared to TritonX-100. To do so, we evaluated the decellularization efficiency using H&E staining and DNA quantification. We also used ELISA and Western-Blot assays to assess the residual antigen and predict the potential immune response after implantation. Collagen, elastin, and GAGs were quantified and qualitatively analyzed to assess the extent of extracellular matrix damage caused by the decellularization process. In addition, we tested its mechanical properties using a tensile test. To verify any potential cytotoxicity of residual detergent, we conducted an *in vitro* cytotoxicity test. Finally, we carried out a rat subcutaneous embedding test to evaluate the biocompatibility and immune response after implantation, providing valuable insights for further clinical applications.

## 2 Materials and methods

### 2.1 Materials

The bovine pericardium is obtained from the local slaughterhouse. After taking out the sample, immerse it in pre-cooled phosphate-buffered saline (PBS) solution for storage and transport it to the laboratory within 4 h. Check for any obvious knife injuries with a magnifying glass, then remove all the areas that do not meet the requirements. Carefully remove adipose tissue on the material matrix with scissors and tweezers, and clamp the tweezers parallel to the material to avoid damaging it. After treatment, store it at 4 °C for the next experiment.

### 2.2 Decellularization treatment

Fresh bovine pericardium tissues underwent treatment using three distinct methods: (a) 3% AES, (b) 1% AES, and (c) 0.25% Triton X-100 (Sigma, X-100, United States). Specifically, fresh pericardium tissues were incubated separately with 1% or 3% AES, with shaking at 100 rpm for 24 h at 37°C, or alternatively, treated with 0.25% Triton X-100 for 48 h under identical conditions. The pericardium tissues were then rinsed thrice with the sterile PBS solution for 10 min and shaken. Next, they were treated with DNase (3U/ml), RNase (0.03 mg/ml), (2.5 mm) Mg2+, and (0.1 mm) Ca2+ for 24 h at 37°C, with shaking at 100 rpm. Finally, the pericardium tissues were rinsed with the sterile PBS solution for 24 h to ensure complete cleanliness.

### 2.3 Light microscopy

All pericardia were fixed in formaldehyde, processed, embedded in paraffin, and sectioned at 5 μm for subsequent light microscopy analysis. Hematoxylin and eosin (H&E), elastic van Gieson (EVG), alcian blue, and Masson’s trichrome staining were employed to visualize and assess the cell nuclei (appearing blue-black), elastin network (appearing black), glycosaminoglycans (appearing sky blue), and collagen fibers (appearing blue), respectively.

### 2.4 Quantitative chemical analyses

To quantify the DNA content of each sample, as well as the three main components of the ECM, including collagen, elastin, and GAGs, a DNA detection kit, hydroxyproline detection kit (Nanjing Construction Engineering Group a030-two to one, China), DMMB detection kit (genmed gms19239.2, United States), and Elastin detection kit (Biocolor United Kingdom) were utilized. The instructions provided with each kit were followed for details. In total, six samples were tested in each group.

### 2.5 Anti-native bovine pericardium serum production

The protocol outlined by Leigh G. Griffiths (Wong et al., 2013) was used to generate anti-BP serum. All procedures were conducted in compliance with the animal care and use guidelines of the Second Xiangya Hospital of Central South University. Specifically, New Zealand white rabbits (n = 6) were immunized with homogenized BP, and serum was collected after 84 days and stored at −80°C.

### 2.6 Protein extraction

Manual mincing of all pericardia was performed, followed by incubation in a protein extraction solution containing 0.1% sodium dodecyl sulfate (SDS) at 1000 rpm and 4°C for 1 h. The supernatant was then collected after centrifugation, and all extracts were stored at −80°C.

### 2.7 Residual antigen detection

To detect the antigenicity of protein extracts, ELISA was performed. The antigen removal rate was determined using an anti-native bovine pericardial serum. Murine anti-Gala1-3Gal b1-(3)4GlcNAc-R (alpha-gal) (Enzo Life Sciences, ALX-801-090, Farmingdale, America) and Anti-MHC-1 Antibody Kit (Bio-Rad AbD Serotec, MCA244GA, Hercules, America) were selected to detect all sample proteins. Protein extraction solution volume served as loading control for all western blot images, with equal volumes loaded in each well to ensure equal starting tissue mass per well (n = 3 per group) (Dalgliesh et al., 2018). Densitometry was determined using ImageJ acquisition and analysis software, with all lanes corrected for background. The antigen expression levels were based on native bovine pericardium used as the baseline, and the ratio of the remaining samples was calculated.

### 2.8 Thermal stability testing

The samples from each group were cut into strips of 1 cm × 5 cm (n = 5) for thermal stability testing. Distilled water was used as the medium and the samples were heated, starting from 20°C and increasing the temperature by 5°C per minute. The shrinkage temperature was measured using an HG-1 leather shrinkage temperature tester (Sichuan Chengdu Dachengxing Digital System Co., Ltd.).

### 2.9 Mechanical testing and thickness measurement

Each group of materials was cut into strips of 1 cm × 5 cm to perform mechanical tests. The thickness and tensile length of each sample were measured using an electronic tensile tester (Instron, United States). At a tensile rate of 100 mm/min, the elastic modulus (MPa), ultimate tensile stress (MPa), maximum load (N), and strain failure (%) of the material was calculated.

To measure the thickness, fresh bovine pericardium of similar thickness was cut into 3 cm × 3 cm, and the thickness of five points (four corners and core) was measured using a thickness gauge (ID-C112XB, Mitutoyo, Neuss, GER) after decellularization. The average of the five points was adopted as the thickness data.

### 2.10 Cell culture and cytotoxicity assessment

The human umbilical vein endothelial cell line (EAhy926) was cultured in high glucose Dulbecco’s Modified Eagle Medium with 10% fetal bovine serum (DMEM/10%FBS). Samples of 0.5 cm × 0.5 cm were cut from all samples and cultured in DMEM/10%FBS at 37°C for 24 h at a density of 2.5 ml/cm^2^. The culture media (leach liquor, the co-culture solution of each sample) was collected and preserved. The media was replaced with leach liquor from the sample cultures diluted with DMEM/10%FBS at ratios of 1:2. The cells were cultured for further 1, 3, and 5 days at 37°C. A negative control was prepared using DMEM/10%FBS alone.

The mitochondrial metabolic (MTT) assay was used to assess cell growth in the samples. The optical density at 570 nm was determined using a microplate reader. The cytotoxicity of each protocol was evaluated by calculating the relative growth rate (RGR, RGR = (mean OD for each group)/(mean OD of the negative control)*100%) to determine the proliferation index (XU et al., 2017).

### 2.11 Subcutaneous implantation in rats

The animal experiments were conducted following ethical guidelines approved by the Second Xiangya Hospital of Central South University Animal Experiment Ethics Committee and Authority for Animal Protection. The ARRIVE guidelines were strictly adhered to, and the study was carried out in accordance with the United Kingdom Animals (Scientific Procedures) Act, 1986, and its associated guidelines. In this study, SD rats (4-5 weeks old, male, 80–120 g) were used for subdermal implantation. Each rat was randomly implanted with one group of samples (n = 3), and the incisions were closed using polypropylene 3-0 sutures. All rats were euthanized on the 14th, 28th, and 56th day using the de-neck method, and the specimens were stored at -80°C for subsequent experiments.

### 2.12 Histological and immunological analysis

The samples were dehydrated, embedded in paraffin, and sliced with a thickness of 5 µm for staining. H&E staining was performed to evaluate cell infiltration and fibrosis. The identification of different types of collagen fibers and their content was determined by Picro-Sirius red staining, which uses polarized light to differentiate between typeⅠand type Ⅲ collagen fibers. TypeⅠcollagen fibers appear yellow/orange, while type Ⅲ collagen fibers appear green under polarized light. Using Image Pro Plus 6.0 analysis software, the pixel area of type Ⅲ collagen in each group was measured uniformly with pixel area as the standard unit.

For immunofluorescence (IF) staining, the sections were incubated with the primary antibody overnight at 4°C. A rabbit anti-rat CD68 antibody (Servicebio Co. Ltd. Wu Han China; dilution 1:500) was used to label macrophages (red), while a rabbit anti-rat CD3 antibody (Abcam; dilution 1:100) was used to label T cells (red).

### 2.13 Recipient graft-specific antibody titer

Homogenized native BP was bound in a 96-well plate and probed with rat sera collected at different time points post-implantation (day 0, 14, 28, and 56). A total of three rats were used per group, per time point. Linear regression of the reference curve (day 0 rat serum) was used to determine the temporal graft-specific production of antibodies. The titer from days 14, 28, and 56 was normalized to day 0 for each rat, resulting in a fold-change relative to the baseline for each rat/time point.

### 2.14 Enzyme-linked immunosorbent assay

Serum levels of TNF-α and IL-6 were measured at 14, 28, and 56 days after implantation to assess the inflammatory response. The levels were determined using enzyme-linked immunosorbent assay (ELISA) according to the manufacturer’s instructions (Wuhan service biotechnology CO Ltd, Wuhan, China; Shanghai FANKEL Industrial CO Ltd, Shanghai, China).

### 2.15 Statistical analyses

Data were expressed as mean ± standard deviation (n ≥ 3). One-way and two-way analysis of variance (ANOVA) followed by Tukey’s *post hoc* test was performed to analyze the data, with statistical significance set at *p*-value<0.05, indicated by asterisks.

## 3 Results

### 3.1 Decellularization efficiency

**FIGURE 1:**
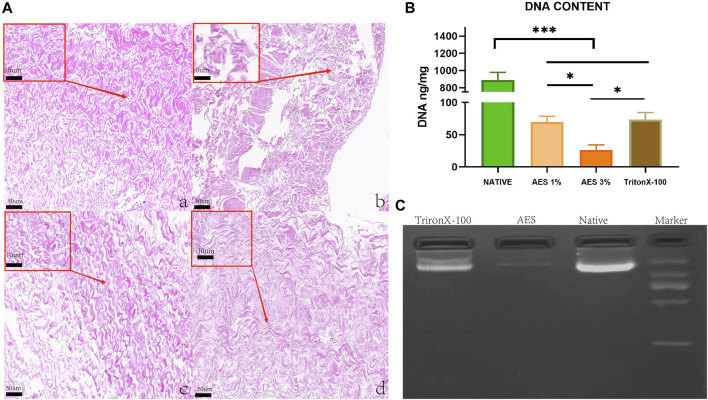
**(A)** H&E staining: **(Aa)** Native, **(Ab)** 1%AES, **(Ac)** 3%AES, **(Ad)** 0.25% TritonX-10, Low magnification (×20); (rectangular box) High magnification (×100). **(B)** DNA content after decellularization; **(C)** DNA ladder.

To assess the effectiveness of decellularization, we utilized H&E staining to visualize the extracellular matrix and cellular removal after the process. As shown in Figure 1A, the extracellular matrix in the 3%AES group (Ac) exhibited a complete structure than the triton group (Ad) (the Ac had a denser extracellular matrix structure and a more regular fibrous alignment without broken fibers), and no nuclei were observed in the 3% AES group. Additionally, we quantitatively evaluated the decellularization efficiency through DNA content analysis. Since the decellularization efficiency of the 1% AES group was lower than that of the 3% AES group, the latter was chosen as the experimental group for further tests.

There was a statistically significant difference in DNA content detection between the natural and decellularized pericardium. As shown in Figure 1B/Table 1, the DNA content of the natural, 3% AES, and triton groups were 890 ± 87.43 ng/mg, 26.23 ± 8.04 ng/mg, and 73.4 ± 10.92 ng/mg, respectively. The significant decrease in DNA content suggests that most of the cells were removed from the pericardial material in the 3%AES group, which is consistent with the H&E staining results. The DNA content difference between the 3% AES and triton group was statistically significant (*p* < 0.05), indicating that AES is more efficient than triton in decellularization.

**TABLE 1:** Cell removal efficiency and mechanical properties of each group.

	Native	AES	TritonX-100
DNA Content	890.26 ± 87.43	26.23 ± 8.04	73.4 ± 10.92
Collagen content (ug/mg)HW	47.23 ± 0.72	35.05 ± 0.56	25.9 ± 0.31
Elastin content (ug/mg)DW	95.62 ± 8.41	44.61 ± 3.65	36.86 ± 2.72
GAG content (ug/mg) HW	15.76 ± 1.54	11.73 ± 0.46	4.93 ± 0.30
Elastic modulus (MPa)	155 ± 13.24	188 ± 54.61	153 ± 21.83
Failure strain (%)	18.11 ± 2.91	18.66 ± 3.24	19.39 ± 2.23
UTS (MPa)	15.29 ± 3.89	20.22 ± 4.07	17.11 ± 3.58
Maximum load (N)	93.32 ± 28.89	92.17 ± 30.13	94.14 ± 21.51
Thickness (mm)	0.53 ± 0.06	0.41 ± 0.05	0.54 ± 0.05
Shrinkage Temperature (°C)	68.8 ± 0.2	69.44 ± 0.25	68.84 ± 0.32

DNA ladder analysis was utilized to evaluate the size and abundance of residual base fragments after decellularization. As shown in Figure 1, the triton group had a large number of base residues after decellularization, with most of them being base fragments larger than 1000 BP. Conversely, there was little base residue in the AES group.

### 3.2 Chemical composition

**FIGURE 2:**
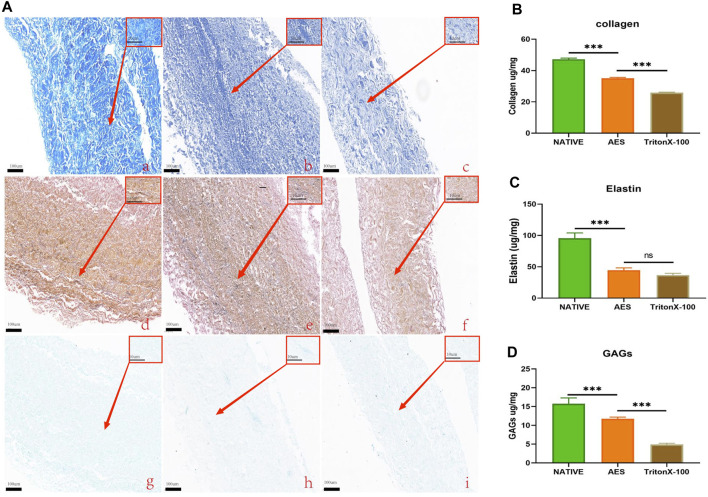
**(A)** Characteristics extracellular matrix after treatment. MASSON staining: **(Aa)**, Native; **(Ab)**, AES; **(Ac)**, triton; EVG staining: **(Ad)**, native; **(Ae)**, AES; **(Af)**, triton; Alcian blue staining: **(Ag)**, native; **(Ah)**, AES; **(Ai)**, triton; Low magnification (×10); (rectangular box) High magnification (×100). Evaluation of collagen **(B)**, elastin **(C)** and GAGs **(D)** content of ECM (n = 6; *p < 0.05; **p < 0.01; ***p < 0.001; ns represents no significant difference).

Masson trichrome and EVG staining were used to assess the extracellular matrix (ECM) morphology of the native, AES, and triton groups. The results showed that the AES group had a more complete and denser ECM compared to the triton group, and there was no obvious breakage of collagen and elastic fibers (Figure 2A).

To quantify the content of the extracellular matrix components, we measured the collagen, elastin, and GAGs contents of three groups. The collagen contents of the native, AES and triton groups were 47.23 ± 0.72 ug/mg, 35.05 ± 0.56 ug/mg, and 25.9 ± 0.31 ug/mg, respectively. Compared to the native group, both decellularization methods resulted in different degrees of collagen loss, with the triton group experiencing more loss than the AES group (*p* < 0.05) (Figure 2B). After decellularization, elastin also experienced some loss, but there was no significant difference between the AES group (44.61 ± 3.65 ug/mg) and the triton group (36.86 ± 2.72 ug/mg) (*p* > 0.05) (Figure 2C). In contrast, the GAGs content in the AES group was significantly higher than that in the triton group (11.73 ± 0.46 ug/mg vs. 4.93 ± 0.30 ug/mg, *p* < 0.05) (Figure 2D).

### 3.3 Antigen removal efficiency

**FIGURE 3:**
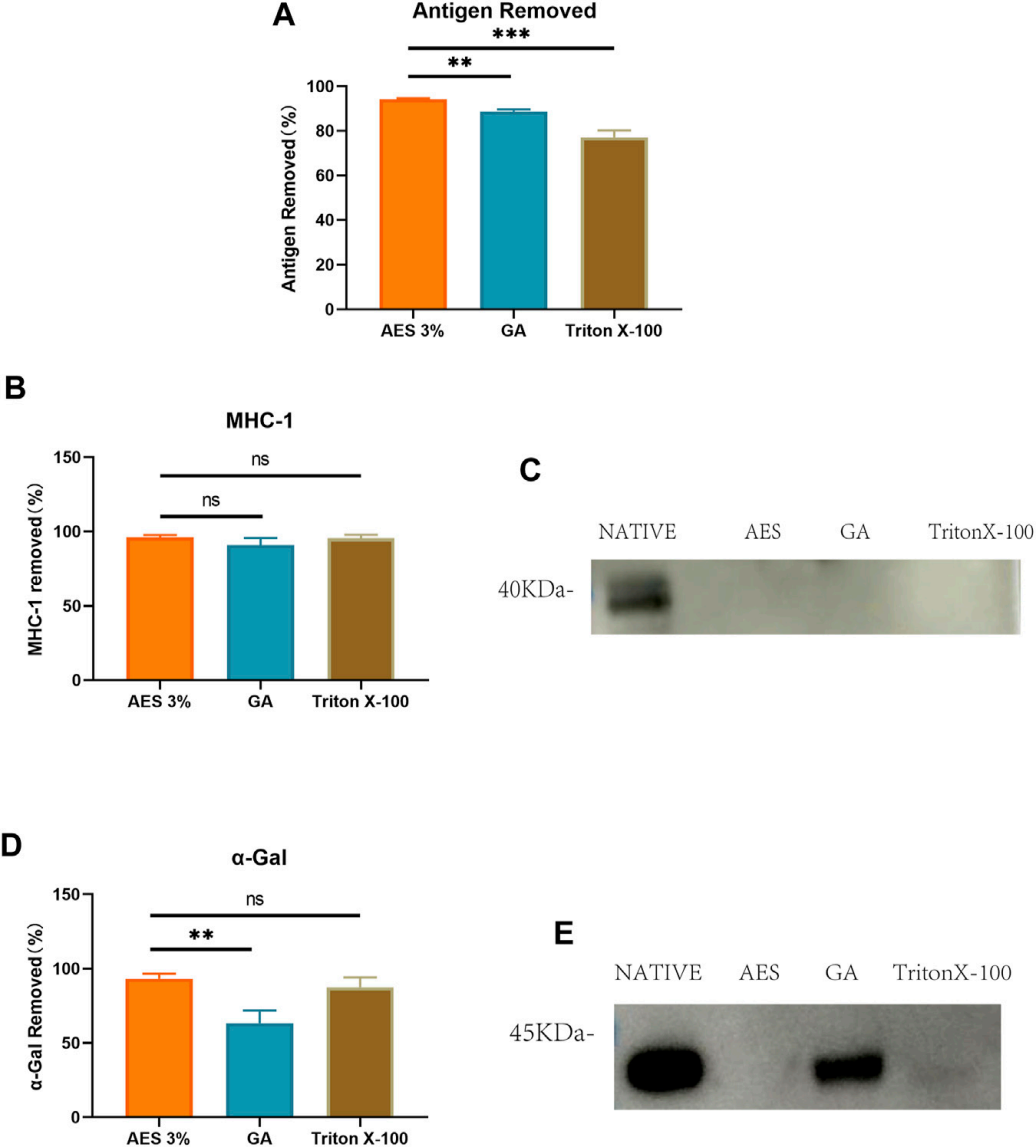
**(A)** Use Elisa to detect antigen removed by AES, GA and TritonX-100. (P.S. GA actually does not remove antigens, but rather seals them). Quantitative comparative analysis of transplantation-associated antigens including MHC-1 **(B)** and α-Gal **(D)**; **(C)** and **(E)** show that the expression of MHC-1 and α-Gal on Western-Blot (n = 3; *p < 0.05; **p < 0.01; ***p < 0.001; ns represents no significant difference).

To compare the effectiveness of different decellularization methods in removing bovine pericardial antigens, we used ELISA to measure the residual antigen content with anti-native bovine pericardial serum. As illustrated in Figure 3, two decellularization methods were found to effectively reduce the antigen content of native bovine pericardium. The average antigen removal rate of the AES group (94.19 ± 0.39%) was significantly higher than that of the Triton group (77.1 ± 3.12%) and Glutaraldehyde (GA) group (88.75.22 ± 0.86%) (note that GA does not remove antigens, but seals them). Transplant-specific antigens can trigger transplantation-related immune responses post-implantation. We employed Western-Blot to compare the efficiency of removing α-Gal and MHC-1 antigens by different decellularization methods. As depicted in Figure 3, compared to the native group, the AES group almost completely removed α-Gal and MHC-1 (93.3 ± 3.43%; 96.03 ± 1.65%).

### 3.4 Mechanical properties

**FIGURE 4:**
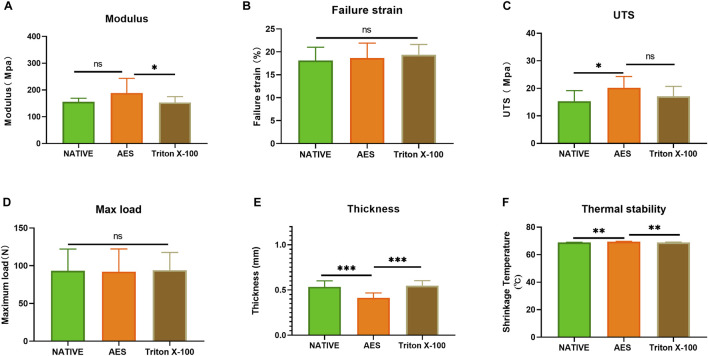
Mechanical performance of each sample **(A)** Young’s modulus; **(B)** Failure strain; **(C)** The ultimate tensile strength (UTS); **(D)** Max load. **(E)** Thickness (n = 12; *p < 0.05; **p < 0.01; ***p < 0.001; ns represents no significant difference); **(F)** shrinkage temperature (n = 5; *p < 0.05; **p < 0.01; ***p < 0.001; ns represents no significant difference).

We conducted mechanical testing on all sample groups. The result of mechanical properties was presented at Table 1/Figure 4, the thickness of the AES group decreased compared to the other groups (native: 0.53 ± 0.06mm; AES: 0.41 ± 0.05mm; Triton X-100: 0.54 ± 0.05 mm). No significant difference (*p* < 0.05) was observed between the AES and native groups in terms of the Young’s modulus and the maximum load. However, the Young’s modulus of the triton group was lower than that of the other two groups. After AES and Triton X-100 decellularization, the failure strain did not change significantly. The ultimate tensile strength (UTS) of the AES group slightly increased, which might be closely related to the fiber arrangement (stress-strain curve shown in Supplementary Figure S1) This finding also suggests that the AES treatment did not significantly affect the mechanical properties.

### 3.5 Thermodynamics stability

We conducted thermal stability testing on each sample to evaluate its ability to withstand high temperatures. The results showed no significant difference between the native group (68.8 ± 0.2°C) and the triton group (68.8 ± 0.3°C) (*p* > 0.05). However, the thermal stability temperature of the AES group (69.44 ± 0.25°C) was slightly increased compared to the native group (*p* < 0.05). These findings suggest that AES treatment did not decrease the overall stability of the pericardium.

### 3.6 Biocompatibility *in vitro*

**FIGURE 5:**
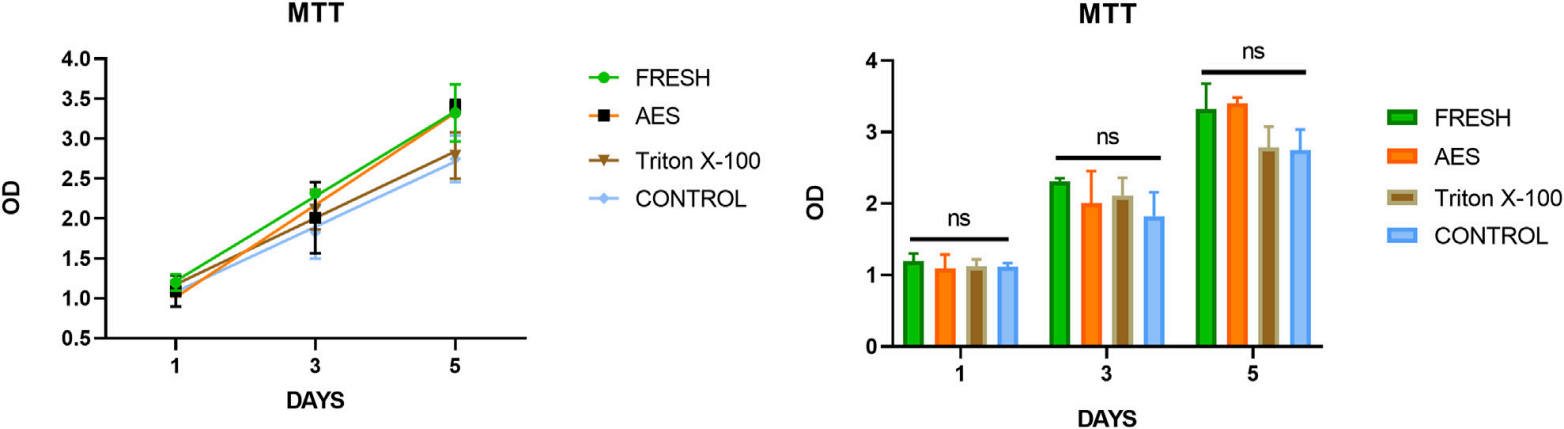
The cytotoxicities were determined by MTT assay, there was insignificance of the relative growth ratio in the experiment groups and control groups (*p* > 0.05).

Residual detergents left on the decellularized pericardium may limit its application due to their potential toxicity to cell regeneration. Therefore, it is crucial to evaluate the potential toxicity after decellularization. To assess residual toxicity, we used the MTT cell proliferation assay to evaluate the relative growth ratios (RGRs) of HUVECs grown in the presence of leach liquor from the sample at different concentrations after 1, 3, and 5 days of culture. As shown in Figure 5, no significant differences in the RGR were observed between all groups on day 1. On day 3 and5, the RGR of all groups exceeded 75%, indicating that the AES group had no significant toxic residue and exhibited excellent biocompatibility (The status of the culture cells shown in Supplementary Figure S2).

### 3.7 *In vivo* biocompatibility and immune response

**FIGURE 6:**
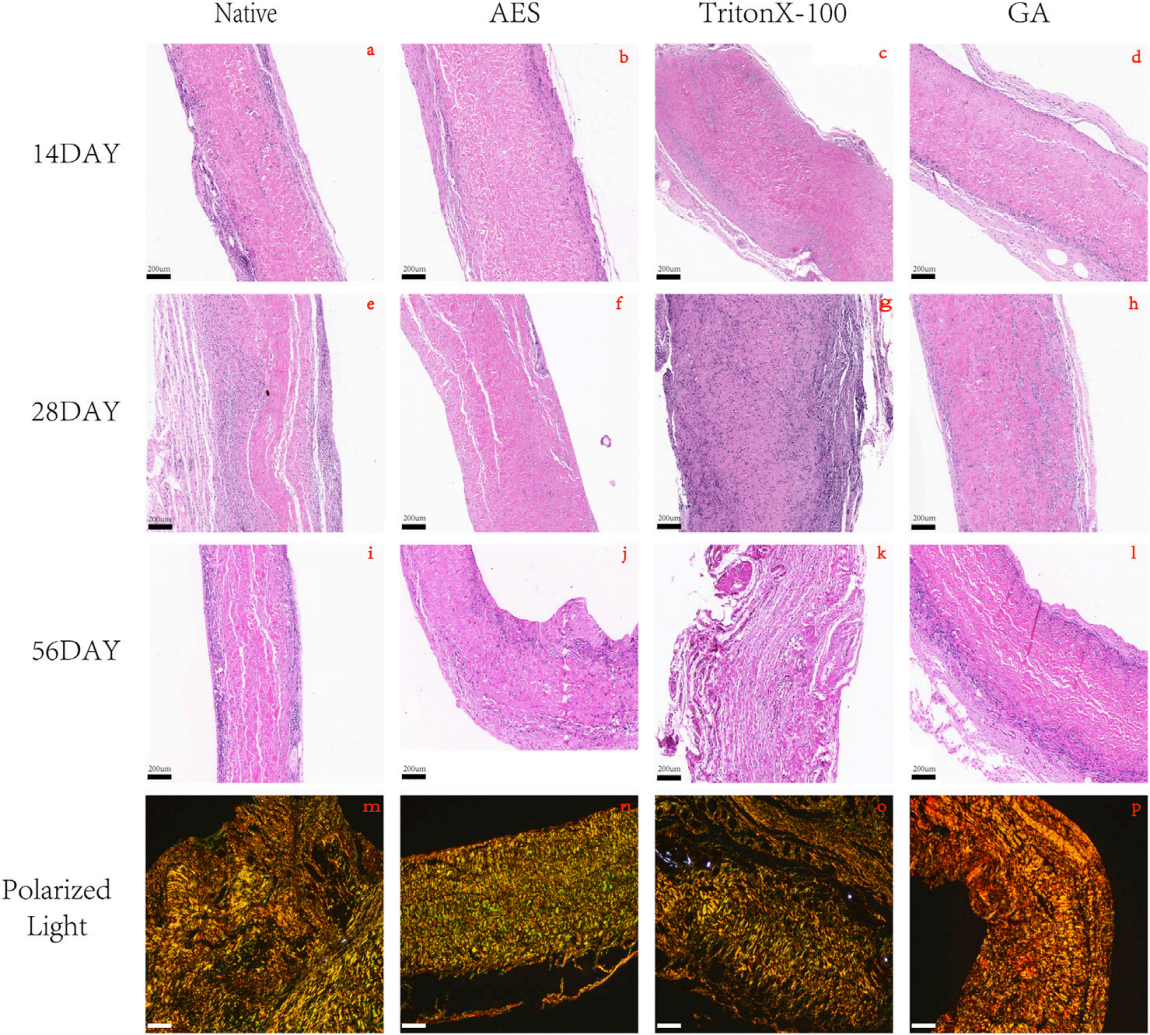
H&E staining after 14 days, 28 days, 56 days of subcutaneous implantation in rats. **(A**, **E**, **I)** Native; **(B**, **F**, **J)** AES; **(C**, **G**, **K)** TritonX-100; **(D**, **H**, **L)** GA, magnification (×4). **(M**, **N**, **O**, **P)** 56days of Picro-sirus red staining: **(M)** Native; **(N)** AES; **(O)** TritonX-100; **(P)** GA, magnification (×10); The color of type Ⅰ collagen fibers is yellow/orange birefringence and type Ⅲ collagen fibers is green birefringence. (Scale bars are 200 um).

**FIGURE 7:**
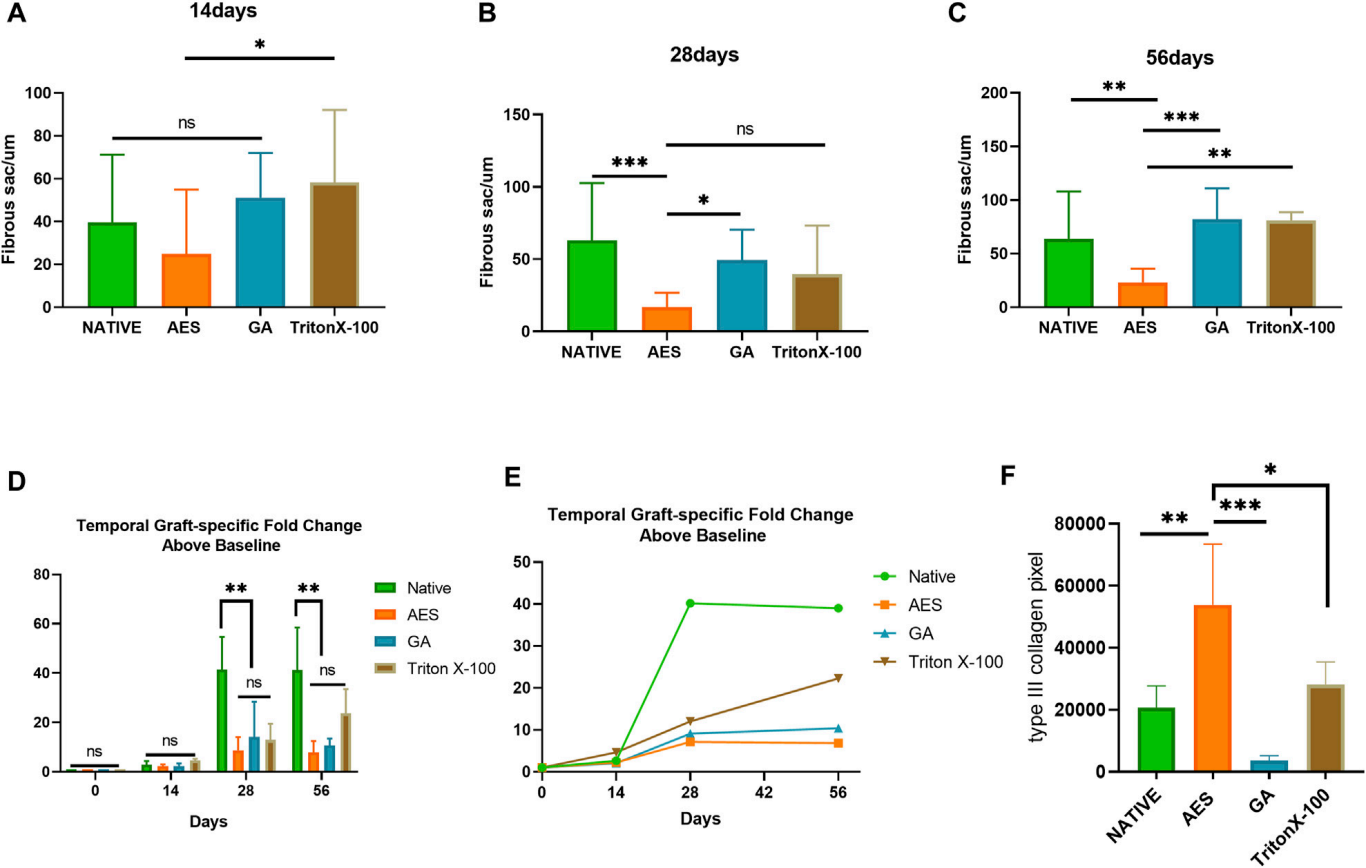
Fibrous sac and graft-specific humoral immune response of each sample. Fibrous sac thickness of each group in 14 days **(A)**, 28 days **(B)**, 56 days **(C)** (n = 15; *p < 0.05; **p < 0.01; ***p < 0.001; ns represents no significant difference); **(D**, **E)** Temporal Graft-specific Fold Change Above Baseline of Native, AES, GA, TritonX-100. (n = 3; *p < 0.05; **p < 0.01; ***p < 0.001; ns represents no significant difference); **(F)** pixel area of type Ⅲ collagen by Picro-Sirius red staining. (n = 4; *p < 0.05; **p < 0.01; ***p < 0.001).

In order to assess the *in vivo* biocompatibility and immune response, H&E staining was conducted at 14 days, 28 days, and 56 days after subcutaneous implantation, as shown in Figure 6. The degree of cell infiltration was observed to gradually increase over time, with complete fiber sac formation occurring around the embedded samples in each group by day 14. Analysis of Figure 7 revealed no significant difference in fiber sac thickness among the four groups on day 14. However, by day 28 and 56, the AES group showed significantly lower fiber sac thickness than the other three groups, which indicates that the inflammatory response of the AES group was lighter, and the AES group may have stronger regeneration ability compared to the other three groups (Zheng et al., 2005).

Cell infiltration is an important indicator of *in vivo* biocompatibility. Compared to the native group, the AES group showed deeper cell infiltration, almost reaching the full layer, which provides evidence for better *in vivo* biocompatibility in group AES. Notably, the triton group exhibited substantial degradation of the embedded tissues by day 56, indicating the most severe local inflammatory reaction, which is consistent with the antibody titer level of the immune response.

Collagen regeneration after subcutaneous implantation is indicative of the regeneration performance of biomaterials (Kawecki et al., 2018). Picro-Sirius red staining was used to assess collagen regeneration in each group, with the pixel area type Ⅲ collagen being highest in the AES group and lowest in the GA group. This implies that the AES group has the strongest regenerative ability.

**FIGURE 8:**
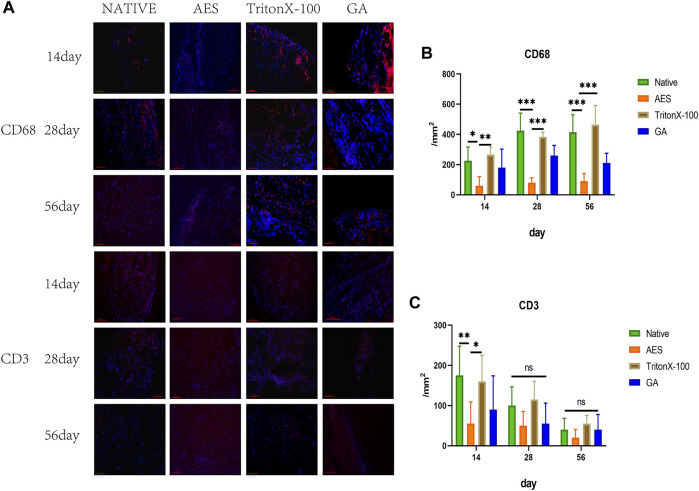
**(A)** Immunofluorescence staining of 14 days, 28 days, 56 days after subcutaneous implantation, immunofluorescence were used to observe the different types of inflammatory cells marker, CD68^+^ Macrophages (red) scale bars are 100 um, CD3^+^T cell (red) scale bars are 100 um; The number of CD68^+^ macrophages **(B)** and CD3^+^ T cells **(C)** after subcutaneous implantation(n = 5; *p < 0.05; **p < 0.01; ***p < 0.001; ns represents no significant difference).

Immunofluorescence staining (CD68 and CD3) was utilized to evaluate the inflammatory response of each group after subcutaneous implantation on 14, 28, and 56 days. As depicted in Figure 8, CD3^+^ T cells appeared on day 14 and gradually decreased by day 28, being almost impossible to observe by day 56. Quantitative analysis of CD3^+^ showed that AES had the lowest level of CD3^+^. Macrophages are an essential indicator of the inflammatory response, with CD68^+^ macrophages beginning to appear on day 14 and reaching the highest level on day 28. Quantitative analysis showed that the expression of CD68^+^ embedded in the AES group was the lowest among all implanted groups (*p* < 0.05).

### 3.8 Graft-specific humoral adaptive immune response

To determine the sensitivity of the adaptive humoral immune response to residual scaffold antigenicity, graft-specific antibody production was examined. The serum antibody titer before subcutaneous implantation was used as the reference value and set to 1. The antibody titer of the subcutaneously embedded samples in each group gradually increased from 14 days (Figure 7/Table 2). There was a positive correlation between the level of graft-specific antibody titer and the residual amount of antigen. The native group showed the highest antibody titer level at each time point, whereas the AES group had the lowest, indicating a weaker immune response to pericardial implantation. Notably, all groups except for triton reached their highest point at 28 days and then stabilized. However, the antibody titer level in the triton group continued to increase with time, and it still showed an upward trend at 56 days, possibly due to its degradation at this point.

**TABLE 2:** Expression level of graft-specific humoral adaptive immune response in each group.

Scaffold	Day 0	Day 14	Day 28	Day 56
Native	1 ± 0	2.885 ± 1.46	41.46 ± 13.24	41.21 ± 17.20
AES	1 ± 0	2.29 ± 0.66	8.72 ± 5.28	7.85 ± 4.6
GA	1 ± 0	2.27 ± 1.16	14.13 ± 14.23	10.67 ± 2.82
TritonX-100	1 ± 0	4.66 ± 0.61	12.98 ± 6.51	23.68 ± 9.81

### 3.9 Expression levels of tumor necrosis factor-a and Interleukin-6 in serum

**FIGURE 9:**
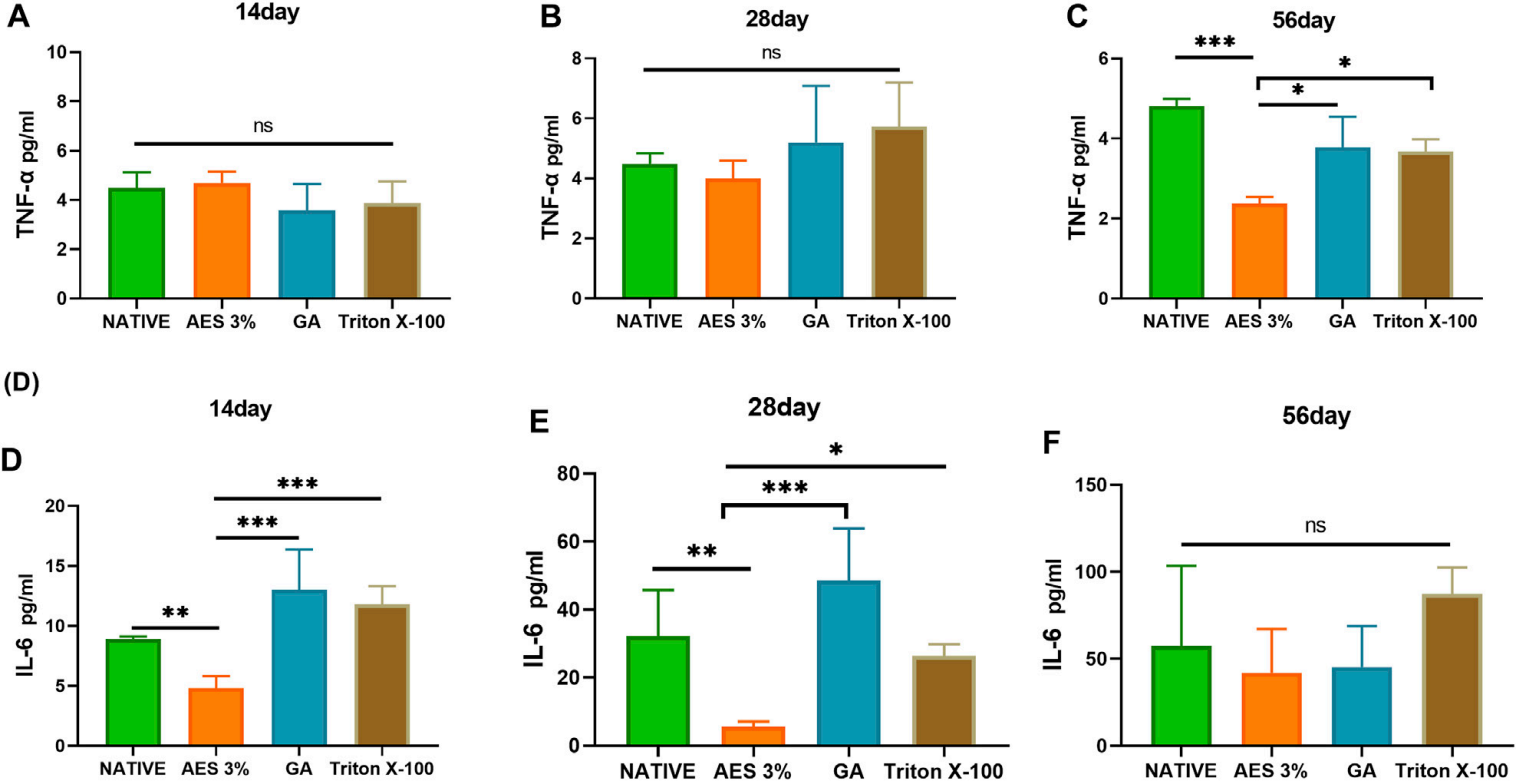
Expression of pro-inflammatory factors in serum in 14 days, 28 days, 56 days. **(A**–**C)** TNF-α **(D**–**F)**, IL-6 (n = 3; *p < 0.05; **p < 0.01; ***p < 0.001; ns represents no significant difference).

To evaluate inflammation-related factors, we measured the expression levels of TNF-α and IL-6 in serum at 14, 28, and 56 days. As shown in Figure 9, there was no significant difference in the expression level of TNF-α between the groups on day14 and 28, while the AES group had the lowest expression level on day 56 (*p* < 0.05).

The expression level of IL-6 increased gradually with the extension of embedding time, and the expression level of each group significantly differed on day 28. The GA group had the highest expression level, and the AES group had the lowest. However, there was no significant difference in the expression level of each group on day 56, likely due to the inflammatory response reaching a plateau.

## 4 Discussion

Decellularization technology has emerged as a critical method for reducing the immunogenicity of biological materials. However, traditional detergents such as Triton X-100 may detrimentally impact the extracellular matrix and only partially eliminate immunogenicity, and Triton X-100 has been withdrawn from the market since 2021 due to its endocrine-disrupting properties, impeding clinical application. To overcome these limitations, we investigated the use of a novel detergent, AES, for the immunogenic removal of bovine pericardium. Concentration is a pivotal factor in the decellularization process, and thus we evaluated two different concentrations of AES alongside 0.25% Triton X-100 as a control to determine the optimal decellularization concentration. Our analyses based on H&E staining and DNA content revealed that 3% AES outperformed both 1% AES and Triton X-100 in terms of decellularization efficiency.

Residual DNA fragments in biological materials are a principal causative factor for host responses post-implantation (Zheng et al., 2005). Consequently, DNA content serves as a critical parameter for assessing decellularization efficiency (Kawecki et al., 2018). Although neither decellularization program could completely eliminate DNA, the 3% AES program removed over 97% of DNA, demonstrating its superior performance. Notably, the length of DNA fragments is a salient determinant of decellularization efficiency, as fragments exceeding 200 bp elicit an immune response (Chakraborty et al., 2020). Our DNA ladder experiment corroborated AES’s superior removal of long base fragments, further highlighting its superior cell removal efficacy.

The immune response triggered by extracellular matrix (ECM) antigens and transplantation-related antigens is a major concern in the use of biological materials for transplantation. In order to evaluate the efficiency of antigen removal, we employed rabbit anti-bovine antibodies to detect the antigen-antibody reaction after treatment with different decellularization reagents. The results indicate that the use of 3% AES can achieve an overall removal efficiency of more than 94% for bovine pericardial materials, whereas the immunogenicity removal efficiency of the triton group was only about 75%. Furthermore, we also evaluated the removal efficiency of two key transplantation-related antigens, namely α-Gal and MHC-1. It was found that AES is more effective in removing α-Gal than glutaraldehyde is in sealing it. These results clearly demonstrate the excellent performance of AES in eliminating bovine pericardium antigens, which is a crucial step toward its clinical application.

The extracellular matrix (ECM) structure is primarily composed of collagen, elastin, and glycosaminoglycans, which play a crucial role in the biomechanical properties and regeneration of heterogeneous tissue materials (Badylak, 2007). We conducted staining observations of collagen, elastin, and glycosaminoglycans on different decellularization treatment schemes. The results show that the ECM structure of the bovine pericardium treated with AES is intact, with collagen and elastin running regularly, while the ECM structure of the pericardium treated with triton is more damaged. We also quantitatively analyzed the contents of collagen, elastic fiber, and glycosaminoglycan. Although decellularization leads to varying degrees of ECM component loss, AES treatment results in less loss of collagen and GAGs. The better preservation of the ECM suggests that AES treatment has a better regeneration effect (Dziki et al., 2017). After valve implantation, the biomaterial needs to undergo long-term blood flow impact, requiring good mechanical properties. Although some ECM components may be lost during decellularization, a direct correlation between ECM content and mechanical properties has not been established. The tensile strength of bovine pericardium was evaluated by the uniaxial tensile test, showing no significant difference between the decellularization group and the native group, indicating that decellularization does not cause a loss of mechanical properties or material durability.

Residual detergents can be potentially cytotoxic (Xu et al., 2014). After AES treatment, cell activity was not significantly lower than that of the native group and did not show obvious cytotoxicity, which may facilitate re-cellularization *in vivo*.

Although the immune response to whole organ transplantation has been studied in detail, the relationship between the immune response to decellularization biomaterials *in vivo* and the degree of antigen removal *in vitro* has not been discussed. We conducted subcutaneous embedding experiments in rats to detect the expression level of the graft-specific humoral adaptive immune response. The results of the antibody expression level suggest that there is a correlation between the degree of antigen removal *in vitro* and the expression of immune response *in vivo*. The more thorough the antigen removal *in vitro*, the lower the immune response *in vivo*. On the 14th day of embedding, there was almost no difference in the antibody expression level among the groups, but by the 28th day, the antibody titer produced by the different embedded samples was significantly different, and almost all reached their peak at this time point. It is important to note that the antibody expression level of the Triton group continued to rise after 28 days, which may be related to the continuous degradation caused by tissue structure destruction. This finding points out the importance of complete antigen removal without causing significant destruction to the original extracellular matrix structure.

The inflammatory response and cell compatibility after implantation are crucial factors in determining a material’s regeneration ability (Dziki et al., 2017; Mahon et al., 2020). The AES group demonstrated favorable performance at different time points after implantation, with its fibrous sac being thinner than the native and Triton groups, suggesting better regeneration possibilities after decellularization. Collagen regeneration is an important indicator of overall regeneration ability (Badylak, 2007). By analyzing the pixel area of type Ⅲ collagen, which is considered to be regenerative collagen (Liu et al., 2022), we conclude that the AES group has better collagen regeneration ability than the other groups.

Upon implantation of biomaterials into the body, inflammatory cells infiltrate and adhere, leading to the secretion of inflammatory factors that severely impact material durability. To address this issue, we analyzed the expression of inflammation-related cells, including CD3^+^ T cells and macrophages. Interestingly, we observed lower macrophage and CD3^+^ T cell expression in samples treated with AES compared to TritonX-100 at each time point after embedding. This finding suggests that AES treatment results in a milder inflammatory response and potentially improved durability, which is consistent with our material degradation results. The inflammatory factors TNF-α and IL-6 are closely related to the inflammatory response and the secretion and development of macrophages (Chakraborty et al., 2020). Therefore, we measured the serum expression levels of IL-6 and TNF-α, and found that the AES group exhibited the lowest levels of inflammatory factors, further supporting our macrophage and inflammatory response results.

It is worth noting that our *in vivo* graft-specific humoral adaptive immune response and inflammatory response are also somewhat consistent, indicating that transplantation-related immune response may impact the generation and development of the local inflammatory response to some extent.

Despite these promising findings, AES comes with limitations. For instance, loss of elastin may lead to calcification after implantation, and we have yet to test the anti-calcification effect. To address this issue, we plan to conduct further experiments to test the anti-calcification effect after antigen removal. This study also has its limitations. Graft-associated antigen assays are initially evaluated *in vitro* using commercially available kits. Subsequent *in vivo* studies typically require the use of knockout animal models to fully elucidate their clinical significance. Owing to the restricted number of samples used in some of the evaluations, the current study can be regarded as a preliminary investigation. Thus, further investigations with larger sample sizes are warranted to corroborate and expand upon the obtained results.

In summary, decellularization is a crucial step before biomaterial implantation, and complete antigen removal is essential. We have demonstrated that the novel detergent AES effectively removes bovine pericardium antigens and results in a mild immune response after implantation. Thus, bovine pericardium treated with AES is an immunologically-acceptable xenogeneic biomaterial that undergoes pro-regenerative host repopulation and remodeling response.

## 5 Conclusion

In this study, a novel detergent AES was used to remove the immunogenicity of bovine pericardium, which can fully remove the antigen components of cells and extracellular matrix without affecting the structure and mechanical strength of the extracellular matrix, and there is no obvious toxic residue. *In vivo*, bovine pericardium treated with AES had a lower level of graft-specific humoral adaptive immune response, less expression of inflammatory response and better possibility of regeneration. Our findings suggest that the treatment of bovine pericardium with AES is a promising method for reducing the immunogenicity of biomaterials compared with traditional decellularization methods. This could lead to the development of more effective cardiac biological valves.

## Data Availability

The original contributions presented in the study are included in the article/Supplementary Material, further inquiries can be directed to the corresponding author.

## References

[B1] Badylak S. F. Gilbert T. W. (2008). Immune response to biologic scaffold materials. Semin. Immunol. 20 (2), 109–116. 10.1016/j.smim.2007.11.003 18083531 PMC2605275

[B2] Badylak S. F. (2007). The extracellular matrix as a biologic scaffold material☆. Biomaterials 28 (25), 3587–3593. 10.1016/j.biomaterials.2007.04.043 17524477

[B3] Cascalho M. Platt J. L. (2001). The immunological barrier to xenotransplantation. Immunity 14 (4), 437–446. 10.1016/s1074-7613(01)00124-8 11336689

[B4] Chakraborty J. Roy S. Ghosh S. (2020). Regulation of decellularized matrix mediated immune response. Biomater. Sci. 8 (5), 1194–1215. 10.1039/c9bm01780a 31930231

[B5] Chang Y. Tsai C. C. Liang H. C. Sung H. W. (2002). *In vivo* evaluation of cellular and acellular bovine pericardia fixed with a naturally occurring crosslinking agent (genipin). Biomaterials 23 (12), 2447–2457. 10.1016/s0142-9612(01)00379-9 12033592

[B6] Chaux A. Gray R. J. Stupka J. C. (2016). Anticoagulant independent mechanical heart valves: Viable now or still a distant holy grail [J]. Ann. Transl. Med. 4 (24), 525. 10.21037/atm.2016.12.58 28149886 PMC5233483

[B7] Collatusso C. Roderjan J. G. De Noronha L. (2019). Decellularization as a method to reduce calcification in bovine pericardium bioprosthetic valves [J]. Interact. Cardiovasc Thorac. Surg. 29 (2), 302–311. 10.1093/icvts/ivz041 30848795

[B8] Crapo P. M. Gilbert T. W. Badylak S. F. (2011). An overview of tissue and whole organ decellularization processes. Biomaterials 32 (12), 3233–3243. 10.1016/j.biomaterials.2011.01.057 21296410 PMC3084613

[B9] Dalgliesh A. J. Parvizi M. Lopera-Higuita M. Shklover J. Griffiths L. G. (2018). Graft-specific immune tolerance is determined by residual antigenicity of xenogeneic extracellular matrix scaffolds. Acta Biomater. 79, 253–264. 10.1016/j.actbio.2018.08.016 30130615 PMC6349227

[B10] Daly K. A. Stewart-Akers A. M. Hara H. Ezzelarab M. Long C. Cordero K. (2009). Effect of the αGal epitope on the response to small intestinal submucosa extracellular matrix in a nonhuman primate model. Tissue Eng. Part A 15 (12), 3877–3888. 10.1089/ten.tea.2009.0089 19563260

[B11] Deng Q. Li H. Sun H. Sun Y. Li Y. (2016). Hyperbranched exopolysaccharide-enhanced foam properties of sodium fatty alcohol polyoxyethylene ether sulfate. Colloids Surf. B Biointerfaces 141, 206–212. 10.1016/j.colsurfb.2016.01.050 26852104

[B12] Dziki J. L. Huleihel L. Scarritt M. E. Badylak S. F. (2017). Extracellular matrix bioscaffolds as immunomodulatory biomaterials. Tissue Eng. Part A 23 (19-20), 1152–1159. 10.1089/ten.tea.2016.0538 28457179 PMC6112165

[B13] Gates K. V. Dalgliesh A. J. Griffiths L. G. (2017). Antigenicity of bovine pericardium determined by a novel immunoproteomic approach. Sci. Rep. 7 (1), 2446. 10.1038/s41598-017-02719-8 28550302 PMC5446425

[B14] Goldstein S. (2005). Decellularization of bovine pericardium for tissue-engineering by targeted removal of xenoantigens. J. heart valve Dis. 14 (5), 705. ; author reply. 16245515

[B15] Hulsmann J. Grun K. El Amouri S. Barth M. Hornung K. HolzfuB C. (2012). Transplantation material bovine pericardium: Biomechanical and immunogenic characteristics after decellularization vs. glutaraldehyde-fixing. Xenotransplantation 19 (5), 286–297. 10.1111/j.1399-3089.2012.00719.x 22978462

[B16] Human P. Zilla P. (2001). Characterization of the immune response to valve bioprostheses and its role in primary tissue failure. Ann. Thorac. Surg. 71 (5), S385–S388. 10.1016/s0003-4975(01)02492-4 11388230

[B17] Jana S. Lerman A. (2015). Bioprinting a cardiac valve. Biotechnol. Adv. 33 (8), 1503–1521. 10.1016/j.biotechadv.2015.07.006 26254880

[B18] Kawecki M. Abu W. Klama-Baryla A. Kitala D. Kraut M. Glik J. (2018). A review of decellurization methods caused by an urgent need for quality control of cell-free extracellular matrix' scaffolds and their role in regenerative medicine. J. Biomed. Mater Res. B Appl. Biomater. 106 (2), 909–923. 10.1002/jbm.b.33865 28194860

[B19] Kostyunin A. E. Yuzhalin A. E. Rezvova M. A. Ovcharenko E. A. Glushkova T. V. Kutikhin A. G. (2020). Degeneration of bioprosthetic heart valves: Update 2020. J. Am. Heart Assoc. 9 (19), e018506. 10.1161/JAHA.120.018506 32954917 PMC7792365

[B20] Liao J. Joyce E. M. Sacks M. S. (2008). Effects of decellularization on the mechanical and structural properties of the porcine aortic valve leaflet. Biomaterials 29 (8), 1065–1074. 10.1016/j.biomaterials.2007.11.007 18096223 PMC2253688

[B21] Liu Y. Chen C. Xie X. Yuan H. Tang Z. Qian T. (2022). Photooxidation and pentagalloyl glucose cross-linking improves the performance of decellularized small-diameter vascular xenograft *in vivo* . Front. Bioeng. Biotechnol. 10, 816513. 10.3389/fbioe.2022.816513 35402413 PMC8987116

[B22] Luo W. Hickman D. Keykhosravani M. Wilson J. Fink J. Huang L. (2020). Identification and characterization of a Triton X-100 replacement for virus inactivation. Biotechnol. Prog. 36 (6), e3036. 10.1002/btpr.3036 32533632

[B23] Mahon O. R. Browe D. C. Gonzalez-Fernandez T. Pitacco P. Whelan I. T. Von Euw S. (2020). Nano-particle mediated M2 macrophage polarization enhances bone formation and MSC osteogenesis in an IL-10 dependent manner. Biomaterials 239, 119833. 10.1016/j.biomaterials.2020.119833 32062479

[B24] Manji R. A. Ekser B. Menkis A. H. Cooper D. K. (2014). Bioprosthetic heart valves of the future. J. Xenotransplantation 21 (1), 1–10. 10.1111/xen.12080 PMC4890621 24444036

[B25] Manji R. A. Zhu L. F. Nijjar N. K. Rayner D. C. Korbutt G. S. Churchill T. A. (2006). Glutaraldehyde-fixed bioprosthetic heart valve conduits calcify and fail from xenograft rejection. Circulation 114 (4), 318–327. 10.1161/circulationaha.105.549311 16831988

[B26] Mirsadraee S. Wilcox H. E. Korossis S. A. Kearney J. N. Watterson K. G. Fisher J. (2006). Development and characterization of an acellular human pericardial matrix for tissue engineering. Tissue Eng. 12 (4), 763–773. 10.1089/ten.2006.12.763 16674290

[B27] Roosens A. Somers P. De Somer F. Carriel V. Van Nooten G. Cornelissen R. (2016). Impact of detergent-based decellularization methods on porcine tissues for heart valve engineering. Ann. Biomed. Eng. 44 (9), 2827–2839. 10.1007/s10439-016-1555-0 26842626

[B28] Schenke-Layland K. Vasilevski O. Opitz F. Konig K. Riemann I. Halbhuber K. (2003). Impact of decellularization of xenogeneic tissue on extracellular matrix integrity for tissue engineering of heart valves. J. Struct. Biol. 143 (3), 201–208. 10.1016/j.jsb.2003.08.002 14572475

[B29] Schoen F. J. Levy R. J. (2005). Calcification of tissue heart valve substitutes: Progress toward understanding and prevention. Ann. Thorac. Surg. 79 (3), 1072–1080. 10.1016/j.athoracsur.2004.06.033 15734452

[B30] Simon P. Kasimir M. T. Seebacher G. (2003). Early failure of the tissue engineered porcine heart valve SYNERGRAFT™ in pediatric patients. Eur. J. Cardiothorac. Surg. 23 (6), 1002–1006. 10.1016/s1010-7940(03)00094-0 12829079

[B31] Thampi P. Nair D. Venugopal S. Ramachandra U. (2013). Pathological effects of processed bovine pericardial scaffolds-A comparative *in vivo* evaluation. Artif. Organs 37 (7), 600–605. 10.1111/aor.12050 23452255

[B32] Wong M. L. Wong J. L. Athanasiou K. A. Griffiths L. G. (2013). Stepwise solubilization-based antigen removal for xenogeneic scaffold generation in tissue engineering. Acta biomater. 9 (5), 6492–6501. 10.1016/j.actbio.2012.12.034 23321301

[B33] Xu H. Xu B. Yang Q. Li X. Ma X. Xia Q. (2014). Comparison of decellularization protocols for preparing a decellularized porcine annulus fibrosus scaffold. PLoS One 9 (1), e86723. 10.1371/journal.pone.0086723 24475172 PMC3901704

[B34] Xu S. Lu F. Cheng L. Li C. Zhou X. Wu Y. (2017). Preparation and characterization of small-diameter decellularized scaffolds for vascular tissue engineering in an animal model. Biomed. Eng. online 16 (1), 55. 10.1186/s12938-017-0344-9 28494781 PMC5425976

[B35] Yacoub M. H. Takkenberg J. J. (2005). Will heart valve tissue engineering change the world? [J]. Nat. Clin. Pract. Cardiovasc. Med. 2 (2), 60–61. 10.1038/ncpcardio0112 16265355

[B39] Yang M. Chen C. Z. Wang X. N. Zhu Y. B. Gu Y. J. (2009). Favorable effects of the detergent and enzyme extraction method for preparing decellularized bovine pericardium scaffold for tissue engineered heart valves. J. Biomed. Mater Res. B Appl. Biomater. 91, 354–61. 19507136 10.1002/jbm.b.31409

[B36] Zheng M. H. Chen J. Kirilak Y. Willers C. Xu J. Wood D. (2005). Porcine small intestine submucosa (SIS) is not an acellular collagenous matrix and contains porcine DNA: Possible implications in human implantation. J. Biomed. Mater Res. B Appl. Biomater. 73 (1), 61–67. 10.1002/jbm.b.30170 15736287

[B37] Zhou J. Fritze O. Schleicher M. Wendel H. P. Schenke-Layland K. Harasztosi C. (2010). Impact of heart valve decellularization on 3-D ultrastructure, immunogenicity and thrombogenicity. Biomaterials 31 (9), 2549–2554. 10.1016/j.biomaterials.2009.11.088 20061016

[B38] Zoller U. J. C. P. (2008). Handbook of detergents. part f [J].

