# Supplementary figures and images for: Preliminary study of improving immune tolerance in vivo of bioprosthetic heart valves through a novel antigenic removal method
Source: Front Bioeng Biotechnol. 2023 Mar 27;11:1141247. doi: 10.3389/fbioe.2023.1141247 (PMC10083275; doi:10.3389/fbioe.2023.1141247)

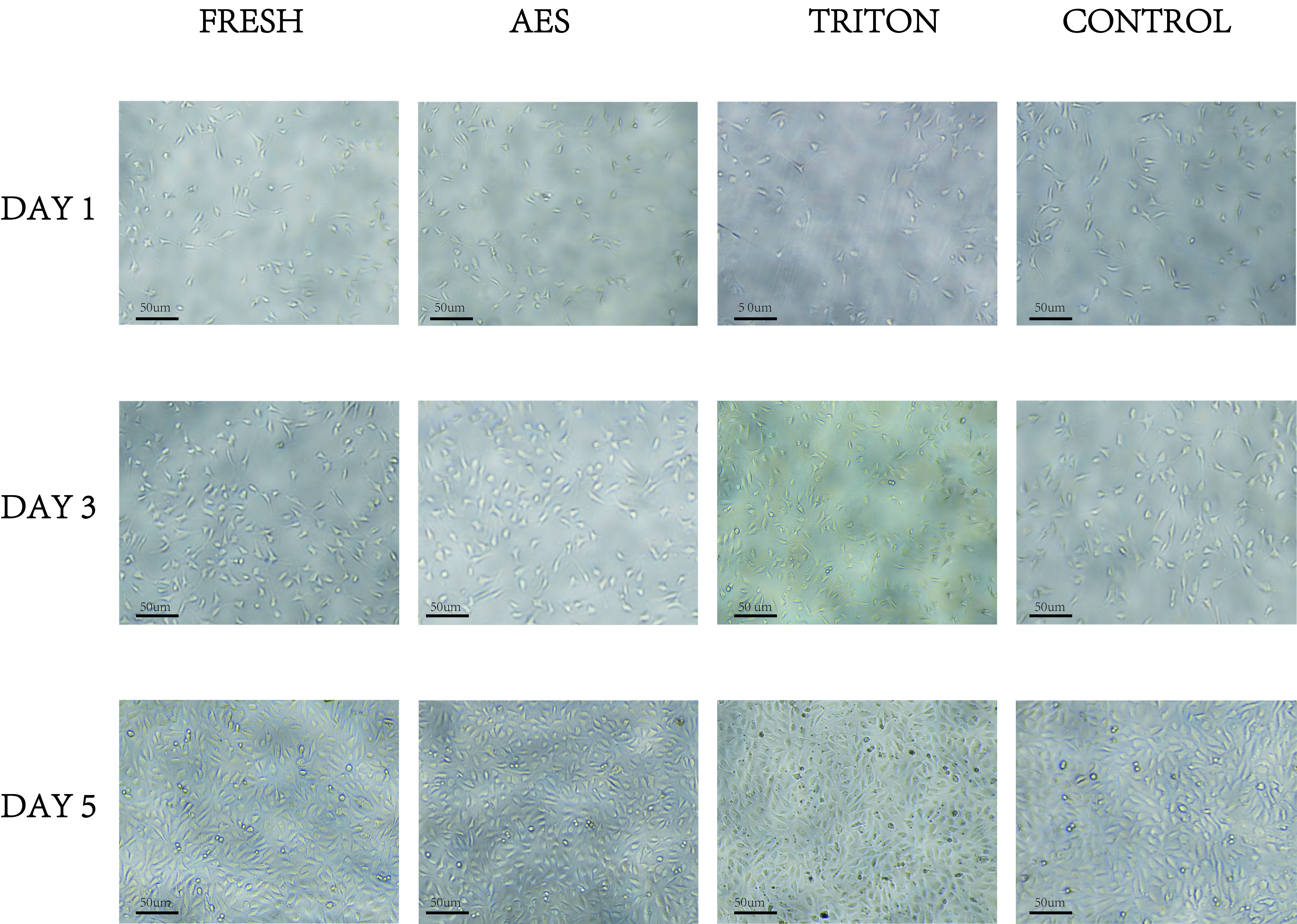

Supplement: Supplementary file 1 [file Image2.JPEG]

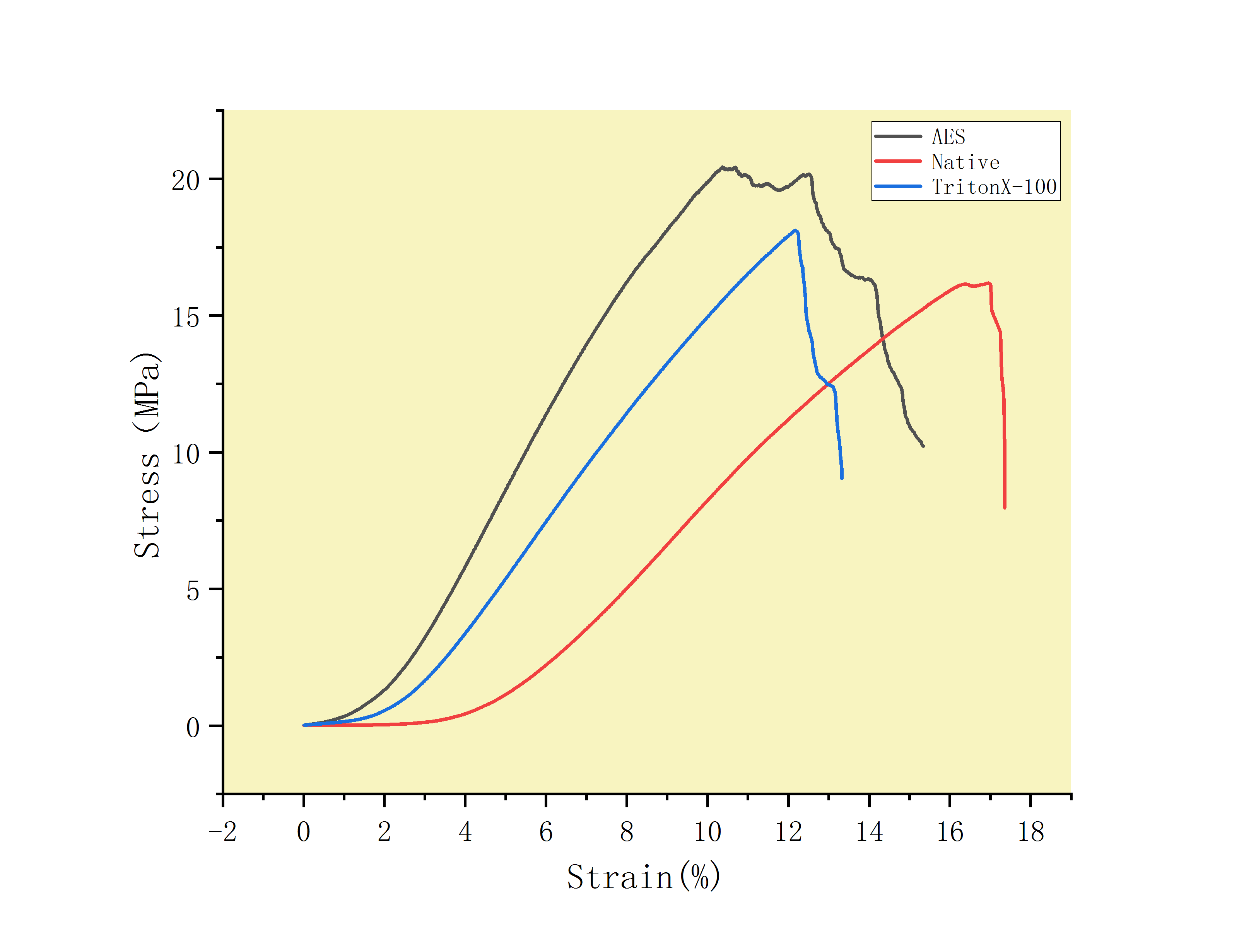

Supplement: Supplementary file 2 [file Image1.TIF]
